# Beyond Clinical Case Studies in Psychoanalysis: A Review of Psychoanalytic Empirical Single Case Studies Published in ISI-Ranked Journals

**DOI:** 10.3389/fpsyg.2017.01749

**Published:** 2017-10-04

**Authors:** Reitske Meganck, Ruth Inslegers, Juri Krivzov, Liza Notaerts

**Affiliations:** ^1^Department of Psychoanalysis and Clinical Consulting, Ghent University, Ghent, Belgium; ^2^Department of Experimental, Clinical and Health Psychology, Ghent University, Ghent, Belgium

**Keywords:** empirical single case studies, psychoanalysis, review, single case archive, psychodynamic psychotherapy

## Abstract

Single case studies are at the origin of both theory development and research in the field of psychoanalysis and psychotherapy. While clinical case studies are the hallmark of psychoanalytic theory and practice, their scientific value has been strongly criticized. To address problems with the subjective bias of retrospective therapist reports and uncontrollability of clinical case studies, systematic approaches to investigate psychotherapy process and outcome at the level of the single case have been developed. Such empirical case studies are also able to bridge the famous gap between academic research and clinical practice as they provide clinically relevant insights into how psychotherapy works. This study presents a review of psychoanalytic empirical case studies published in ISI-ranked journals and maps the characteristics of the study, therapist, patient en therapies that are investigated. Empirical case studies increased in quantity and quality (amount of information and systematization) over time. While future studies could pay more attention to providing contextual information on therapist characteristics and informed consent considerations, the available literature provides a basis to conduct meta-studies of single cases and as such contribute to knowledge aggregation.

## Introduction

Single case studies are at the origin of both theory development and research in the field of psychotherapy in general and psychoanalysis in particular (McLeod, 2010, 2013). Increasingly, empirical case studies made their entrance in the field and are recognized as important sources of evidence to address the complexity of psychotherapeutic processes (Goodheart, 2005; American Psychologcial Association [APA], 2006; McLeod and Elliott, 2011). Systematic meta-studies of single cases moreover allow knowledge aggregation and as such could enhance the scientific merits of case studies (Iwakabe and Gazzola, 2009). We argue that in order to explore the full potential of empirical single cases in the field of psychoanalysis, it is important to map the existing field of such cases and get an overview of their characteristics, strengths and weaknesses. The goal of the current review is to provide this information and delineate points of interest for future case studies and context for meta-studies in the field.

From its origin, the clinical case study was the dominant research method in psychoanalysis. Sigmund Freud, the founding father of psychoanalysis, is still both famous and notorious for his elaborate clinical case studies through which he developed his theoretical framework during the course of his life. Famous because of the richness of his case presentations and because of the resulting theoretical and clinical advancements that up until this day permeate the whole psychotherapeutic and cultural field. Notorious because the scientific merit of this method received increasing criticism and is mostly relegated to the scientific trash can (Bornstein, 2005).

While empirical research continues to be a major source of debate and controversy in psychoanalysis (e.g., Aron, 2012; Benecke, 2014; Mills, 2015), the questioning of its scientific credibility and therapeutic efficacy gave rise to a wealth of (group level) research indicating the efficacy of psychoanalytic psychotherapy (e.g., Leichsenring et al., 2015). Nevertheless, the case study has a privileged place in the field of psychoanalysis. Indeed, the clinical case study is still very common, however, increasingly, empirical case studies appear to see the light of day.

Critiques on the subjective bias of therapists’ retrospective reports, the anecdotal quality and uncontrollable nature of clinical case studies (Spence, 2001) influenced the emergence of systematic (quantitative and qualitative) approaches to conduct single case studies that are no longer (solely) dependent on the interpretation of the therapist. Methodological articles on single-case experimental designs (for a review see Smith, 2012), case-based time series analysis (Borckardt et al., 2008), case comparison methods (Iwakabe, 2011), and theory-building case studies (Stiles, 2008, 2010) are but a few examples of the increased recognition of the potential value of empirical case-based research to build knowledge (Edwards et al., 2004; McLeod and Elliott, 2011). In the entire field of psychology, such idiographic approaches are increasingly considered to be important tools to bridge the science-practitioner gap and address the lack of alignment between the object of study and the method that is often criticized in mainstream evidence-based practice research (e.g., Westen et al., 2004; Desmet, 2013).

Despite this recognition of empirical case study methods, an important critique on generalizability problems remains. A way to address this and build knowledge resulting from single case studies is to conduct meta-studies on published single case studies or as Dattilio et al. (2010, p. 436) state “One observation or one case offers only a small piece of evidence, but repeated observation […] across a series of cases provides a way of constructing a database of evidence on which clinical theory can be built.” Currently the potential for knowledge aggregation across cases surely remains unexplored despite the existence of methodological tools to do so, for example, meta-synthesis methods (Finfgeld, 2003; Willemsen et al., 2015) and case comparison methods (Iwakabe and Gazzola, 2009). A preliminary requirement to facilitate meta-studies, however, is a well-organized database that gathers published case studies (Fishman, 2005; Iwakabe and Gazzola, 2009). The single case archive (SCA; Desmet et al., 2013) provides such a tool for psychoanalytic single cases published in ISI ranked journals.^1^ Such a tool allows gathering cases on a specific topic and conducting meta-studies. Nevertheless, an overview of the nature of existing empirical case studies would provide important contextual information when considering a meta-study, yet this is currently lacking in the literature.

This study attempts to address this need and explores the following questions concerning empirical case studies in the field of psychoanalysis: ‘Which studies have been done?’; ‘What is the nature of these case studies?’; and ‘What are their merits and weaknesses?’ We investigate this through a review of empirical case studies published in ISI-ranked journals.

## Method

Cases were selected through the original SCA (Desmet et al., 2013), which comprises psychoanalytic and psychodynamic case studies, published in ISI-ranked journals between 1955 and 2011. Cases were selected starting from a search on ISI Web of Knowledge using the search terms (psychoanal^∗^ OR psychodynam^∗^) AND (case OR vignette). This search provided 2760 results, which after screening for title, abstract, and if necessary the full text of the article, resulted in 445 articles presenting psychoanalytic or psychodynamic treatment of an original single case (no comments on already published cases). For this study, all English case studies of this dataset that were classified as either experimental (i.e., *N* = 1 subject experiments, testing hypotheses in an experimental design) or (naturalistic) systematic case studies (i.e., case studies using data from sources other than the therapist’s report and where data are investigated by one or more researchers other than the therapist)^2^ were selected (52 articles discussing 55 cases). Moreover, for this study, the same search procedure was followed for the period of 2012–2017 to update the sample with empirical case studies from these more recent years. Screening the 1093 search results resulted in 31 articles discussing 38 empirical cases. All empirical cases were screened with the Inventory for Basic Information in Single Cases (IBISC; Desmet et al., 2013), which inventories basic descriptive information on study characteristics [design, type of data, type of analysis, presence of clinical (process) description, presence of informed consent], therapist (gender, age, education, experience) and patient (gender, age, diagnostic information) characteristics, and therapy characteristics (duration, number of sessions, session frequency, therapy outcome).

## Results

**FIGURE 1:**
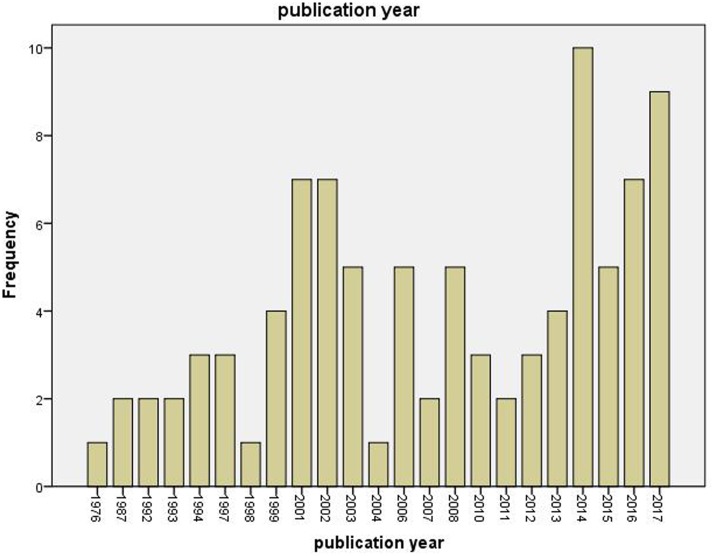
Number of cases per year.

In the screened period (1955–2017), 83 articles were identified that comprise 93 cases using empirical case designs. The selected manuscripts included one to three case studies. **Figure 1** depicts the number of case studies for each year empirical cases could be identified and indicates an increase of published empirical case studies over time.

### Study Characteristics

Of the empirical case studies, five cases (5.4%) were experimental designs and 88 were naturalistic systematic case studies (94.6%) following the definition of Iwakabe and Gazzola (2009). Analysis of the case was mostly based on one type of data (44.1%); 25.8% used two types of data, 14% three types, and 3.2% four or more types of data. The amount of different types of data was not spread equally across time: before 2000 only one study mentioned more than one type of data, between 2000 and 2010 there were regular studies mentioning two types of data; while starting from 2010 studies start to appear that mention three and four or more types of data. The type of data most commonly used were audio recordings (or videotapes) (64.5%), followed by self-report or observer rated scales (36.6%) and interviews (35.5%). In 14% of cases (also) other types of data were used like behavioral measures, notes of the therapist, patient, or relatives, or biological measures.

Different ways of analyzing the data were used, from purely quantitative (21.5%) to purely qualitative (17.2%). Yet, in most studies mixed approaches (56%) were used combining for example clinimetric methods (e.g., ratings of session material using the Shedler Westen Assessment Procedure-200 (SWAP-200; Westen and Shedler, 1999) with clinical, qualitative, or quantitative approaches.

A clinical description of the patient and/or therapy process was provided in 73.1% of the cases. In 24.7% of the cases such a description was lacking, while in two cases there was somewhat of a clinical description through a patients’ retrospective report in one case and a qualitative description of specific analyzed sessions in the other case.

Informed consent (IC) was mentioned in 45.2% of the cases, meaning it was not mentioned in more than half of the cases. Before 2000, IC was mentioned in 4 out of 18 cases, between 2000 and 2010 it was mentioned in 13 out of 35 cases, and between 2011 and 2017 it was mentioned in 25 out of 40 cases. In this last period, of the cases not mentioning IC, four cases did mention an ethical approval, which might indicate that there was also an informed consent on the patient’s side.

### Therapist Characteristics

In most studies information about the therapist was almost entirely lacking. We inventoried information about gender, age, education and experience, yet in 34.4% of the cases there was no information at all. In 10.8% of the cases information about only one of these variables was provided (mostly the therapist’s gender), while in 18.3, 29, and 7.5% of the cases information was provided about 2, 3, and 4 variables respectively.

Concerning gender, 24.7% were female therapists and 36.6% were male therapists. In one case there was both a male and a female therapist involved in therapy. For the remaining 37.8% of cases gender of the therapist was not mentioned. Age was mentioned in only 17.2% of the cases. Education was mentioned in 44.1% of the cases and included all kinds of degrees/description with psychologist, psychotherapist, social worker, and psychiatrist as the most common terms. Experience was mentioned in 57% of the cases, with 10.8% novice therapists (<5 years of experience), 21.5% experienced therapist (6–15 years of experience), and 10.8% senior therapists (>15 years of experience).

### Patient Characteristics

Generally, more information was provided about the patient. There was consistent information about the patients’ gender with 68.8% female and 31.2% male patients. Concerning age, almost always (98.9% of cases) information was present with 6.5% children (2–11 years), 6.5% adolescents (12–17 years), 12.9% young adults (18–24 years), and 73.1% adults (25–65). There were no cases discussing elderly patients.

**FIGURE 2:**
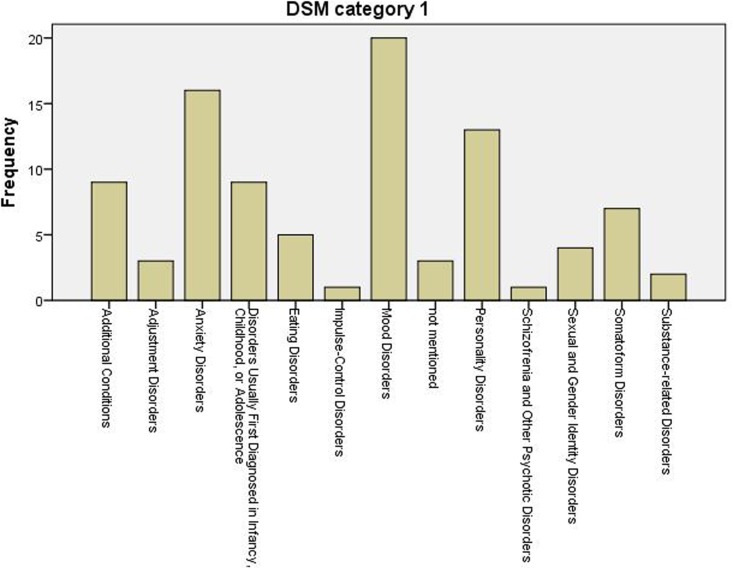
Primary diagnosis of cases per DSM-IV-R category.

There was no information about diagnosis in three cases; all other cases provided diagnostic information. The descriptive terms used in the manuscript are included in the SCA database, however, they differ tremendously across cases. This is illustrated by the observation that in 49.5% of the cases there was no diagnostic system mentioned. In other cases, one or more diagnostic systems were used: 34.5% used a version of the DSM, 10.8% used a version of the ICD, 4.3% used the OPD or PDM, and 8.8% used another system (e.g., SWAP-200, AAI). Therefore, when diagnostic information was available, this was categorized into the main DSM-IV-R categories (multiple categories could apply) to be able to get an overview. Prevalence of diagnostic categories is presented in **Figure 2**. Clearly mood and anxiety disorders were the most common.

### Therapy Characteristics

In considering the characteristics of therapy we see that mostly at least some information is provided about objective characteristics and outcome.

The duration of investigated therapies consisted of 15.1% therapies shorter than 5 months, 12.9% lasting between 6 and 11 months, 32.3% lasting between 1 and 3 years, 18.3% lasting longer than 3 years, and 21.5% of cases were duration was not mentioned. Related to the duration of therapy, the number of sessions was mentioned in 66.7% of cases with 10.8% of therapies comprising less than 20 sessions, 22.6% between 21 and 50 sessions, 12.9% between 51 and 200 sessions, and 20,4% more than 200 sessions.

Session frequency ranged from less than once a week (2.2%), over once a week (35.5%), two to three times a week (17.2%) to a classical analytic setting of four to seven times a week (18.3%) and was not mentioned in 26.9% of the cases.

To inventory therapeutic outcomes the description of the authors was followed and categorized into successful (53.8%), failure (3.2%), or mixed (33.3%). In 9.7% of the cases no information about outcome was provided. Clearly, the description of authors/researchers starts from different frames of reference (e.g., in a long-term analysis success appears to require change at more levels than in studies on short-term psychodynamic psychotherapy who tend to conceptualize success more often as symptom decrease on a symptom rating scale) and consequently any appreciation of outcome is relative to these frames of reference.

## Discussion

Mapping psychoanalytic empirical case studies published in ISI-ranked journals showed that they, while still remaining scarce, clearly increased in quantity and quality throughout the last decennia. The increase in quality is shown in the results of our review that indicate a larger amount of information provided in more recent studies, a broader use of different sources of data and analysis methods, and more explicit informed consent considerations. However, also when reading the articles to rate the IBISC, it was clear that generally there is more attention for a detailed description of the study, its methods, the patient and his or her therapeutic process. Especially, in the last decennium a remarkable increase could be seen in the amount of published cases and the systematic nature of these cases that increasingly include multiple sources of data and combine different methods of analysis. These include both instruments and methods developed within the field of psychoanalysis [e.g., the Core Conflictual Relationship Theme method (Luborsky and Crits-Christoph, 1998) or Reflective Functioning ratings (Bucci, 1997)] and more generic methods that allow the connection to broader psychological research (e.g., the Beck Depression Inventory; Beck et al., 1996).

When considering systematic case studies in the broader field of psychoanalytic case studies – which remain to be mainly clinical case studies – it is clear that they provide much more descriptive information than clinical case studies, which mostly pay little attention to giving comprehensive descriptive information, despite the rich clinical description they provide (Desmet et al., 2013). Nevertheless, information about certain topics remains generally absent. Especially a description of the therapist is often omitted, which might however, be important contextual information if one intends to compare or aggregate different cases. As the role of the therapist in explaining outcome is increasingly recognized (e.g., Kraus et al., 2011) and because of the inherently interactive nature of the therapeutic encounter (e.g., Strupp, 2008), its importance can hardly be overestimated.

The range of diagnoses, short- and long-term therapies, and successful and unsuccessful cases investigated in empirical case studies, however, provide a myriad of possibilities for meta-studies. Moreover, while we noticed a wide array of methods, there also are trends and recurring methods indicating that meta-studies are feasible. For example, methods like Reflective Functioning or the SWAP-200 are used in different studies and should allow for good quality meta-studies. On the other hand, we noticed that studies focusing on purely quantitative methods often omit a clinical description of the patient and the therapy process. In our opinion, this is throwing away the baby with the bathwater. While critiques on the anecdotal nature of clinical case studies may be apt, discarding a clinical or qualitative description altogether is to disown the essence and the strength of the psychoanalytic single case study. Moreover, with respect to clinical relevance and to possible meta-studies, this clinical contextual information is quintessential.

Concerning ethical considerations of research in such delicate circumstances as the psychotherapeutic setting, it appears that even more attention could be paid to informed consent. While informed consent was mentioned much more than was found in the overall archive (Desmet et al., 2013) – where it was mentioned in only 9% of cases – still more than half of the studies did not provide information about informed consent despite their explicit research context.

Surely, our review has certain limitations. Using other search criteria for example, might result in other cases and probably more empirical psychoanalytic cases exist. Also, the inclusion of cases published in books might be a valuable addition. Nevertheless, we think a representative and large sample of systematic case studies could be retrieved through this method. If this is the case, surely future research should aim to investigate age groups that are currently underrepresented in the field as most case studies focus on (young) adults.

We conclude that psychoanalytic empirical case studies, although they were adopted somewhat later than in other orientations, are of increasing and high quality. Moreover, journals currently provide more clear guidelines as to what comprises an eligible case study. High quality cases, for example Gazillo et al. (2014), Mauck and Moore (2014) and Cornelis et al. (2017), set the tone for a future where case aggregation based on scientifically sound cases that include triangulated data and analysis methods while not disregarding clinical context becomes increasingly possible. This attention for the clinical context is of crucial importance in the field of psychotherapy (Spence, 2001). Psychotherapy is and always will consist of a unique encounter between patient and therapist, a complex interaction that cannot be easily disentangled (Strupp, 2008). While systematically investigating what happens in this process is crucial for the advancement of the psychotherapeutic endeavor, this should not come at the cost of the clinical richness of these ever-singular encounters that comprise the magic of the psychotherapeutic profession.

## Author Contributions

RM: conception of the study, contribution to data collection, data-analysis and interpretation, main author of the manuscript; RI: contribution to conception of the study, contribution to data collection, main reviewer of the manuscript; JK: contribution to data collection, data-analysis and interpretation, reviewer of the manuscript; LN: contribution to data collection, data-analysis, reviewer of the manuscript. All authors read and approved the final version of the manuscript.

## Conflict of Interest Statement

The authors declare that the research was conducted in the absence of any commercial or financial relationships that could be construed as a potential conflict of interest.

## Footnotes

1 The original single case archive (www.singlecasearchive.com) comprises 445 articles between 1955 and 2011. Currently, there is an ongoing project to elaborate the SCA with cases from other theoretical orientations that should be finished at the end of 2018.

2 For more information on the types of case studies and their definition, see Iwakabe and Gazzola (2009) or the IBISC manual available through http://www.singlecasearchive.com/downloads/IBISC%20manual3.pdf.
